# Avenues within the gut-liver-brain axis linking chronic liver disease and symptoms

**DOI:** 10.3389/fnins.2023.1171253

**Published:** 2023-07-13

**Authors:** Henry H. Nguyen, Mark G. Swain

**Affiliations:** ^1^University of Calgary Liver Unit, Departments of Medicine and Microbiology, Immunology and Infectious Diseases, Snyder Institute for Chronic Diseases, Cumming School of Medicine, University of Calgary, Calgary, AB, Canada; ^2^University of Calgary Liver Unit, Department of Medicine, Snyder Institute for Chronic Diseases, Cumming School of Medicine, University of Calgary, Calgary, AB, Canada

**Keywords:** microbiome, neural pathways, host immune system, metabolites, sickness behavior, fatigue, depression, social withdrawal

## Abstract

Symptoms of fatigue, social withdrawal and mood disturbances are commonly encountered in patients with chronic liver disease and have a detrimental effect on patient quality of life. Treatment options for these symptoms are limited and a current area of unmet medical need. In this review, we will evaluate the potential mechanistic avenues within the gut-liver-brain axis that may be altered in the setting of chronic liver disease that drive the development of these symptoms. Both clinical and pre-clinical studies will be highlighted as we discuss how perturbations in host immune response, microbiome, neural responses, and metabolites composition can affect the central nervous system.

## Introduction

The liver plays a central role in digestion, metabolism, and immune protection from pathogens (Kalra et al., 2022). Chronic liver disease caused by various etiologies, including viral, metabolic, and autoimmune diseases can impact liver function leading to chronic injury and ultimately liver cirrhosis. The impact of chronic liver injury extends beyond local organ dysfunction, and can cause perturbations systemically affecting extrahepatic organs including the brain (Montano-Loza et al., 2012; Albillos et al., 2014; Liu et al., 2022). This liver-brain interaction can be readily observed in patients who develop hepatic encephalopathy (HE) as a complication of end stage liver disease or liver cirrhosis. Although the pathophysiology of HE is incompletely understood, it is thought that alterations in central nervous system blood flow, accumulation of neurotoxic compounds, presence of inflammatory metabolites, and excess bile acids resulting from poor liver function can mediate Central Nervous System (CNS) dysfunction (Rose et al., 2020). Furthermore, symptoms generated from altered brain function, including fatigue, depression, anxiety, sleep disturbance and loss of social interest have been observed in patients with chronic liver conditions with and without cirrhosis and overall have a detrimental effect on patient quality of life (Swain and Jones, 2019). A more granular understanding of the mechanisms governing changes in brain function in relation to liver health will provide a foundation to further develop new diagnostic and therapeutic tools that can be used to improve the care of liver patients afflicted with these debilitating symptoms. The mechanism that links liver health to brain function is thought to be mediated through various avenues within the gut-liver-brain axis (Ding et al., 2020; Blaga et al., 2021; Brescia and Rescigno, 2021; D’Mello and Swain, 2021). In this review, we will discuss the current understanding of the relationship between liver health and brain function through the lens of both clinical and basic science.

## Host microbiome and immune response

While many different avenues within the liver-brain axis link liver health status with brain function, prior work has highlighted the importance of the host immune response and its associated signaling molecules in impacting brain function (D’Mello and Swain, 2021). While the CNS is often considered an immune privileged site, research suggests that the central nervous system’s (CNS) unique lymphatics and altered blood flow in disease states allow for immune responses to impact the CNS (Louveau et al., 2015). Liver immune cells and the proinflammatory cytokines they produce, including Interleukin 6 (IL-6), Tumor Necrosis Factor Alpha (TNFα) and Interleukin 1 beta (IL-1β), have all been implicated in driving behavioral changes in the setting of liver inflammation. Specifically, these cytokines can affect peripheral neural signaling, be released into circulation and enter the CNS in areas devoid of an intact blood–brain-barrier, alter the blood brain barrier to affect neurons within the brain, or be produced locally within brain tissue via migrating blood mononuclear cells (Kerfoot et al., 2006; D’Mello et al., 2009, 2013). This cytokine-mediated signaling is hypothesized to ultimately impact neurotransmission, particularly within the basal ganglia, leading to altered CNS function in the setting of liver disease (Capuron and Miller, 2011; D’Mello and Swain, 2014; Almishri et al., 2022).

Current evidence strongly suggests that the host immune response can in part be shaped by the host microbiota (Ivanov et al., 2009; McCoy et al., 2017; Zheng et al., 2020). Understandably, the microbiome has therefore also been implicated in driving CNS outcomes through mediating aberrant host immune cell signaling pathways (Kim et al., 2017; Cryan et al., 2020). From the standpoint of liver disease, both clinical and basic science studies have pointed to unique gut microbial signatures being associated with differential liver disease outcomes (Loomba et al., 2017; Albillos et al., 2020; Oh et al., 2020; Acharya and Bajaj, 2021). Although the literature surrounding host microbiome composition being a biomarker for various liver diseases can be heterogeneous, lower diversity, decrease in specific microbes including *Ruminococcaceae, Coprococcus, Faecalibacterium*, *Prevotella,* and microbial-driven changes in gut permeability have all been described in chronic liver disease and clinically implicated in altered brain function in patients (Loomba et al., 2017; Bajaj, 2019; Caussy et al., 2019; Aron-Wisnewsky et al., 2020; Oh et al., 2020; Rose et al., 2020). Mechanistically, it is hypothesized that the microbiome and/or microbial generated metabolites can interact with host cells through a myriad of receptors, in various cellular (both immune and non-immune) contexts, to impart an effect on host health outcomes. Examples of this include microbiota-dependent perturbations in bile acid metabolism, changes in host gut barrier function, and production of metabolites that interact with host receptors, including toll like receptors (e.g., endotoxin) and aryl hydrocarbon receptors (e.g., indoles) (Albillos et al., 2020; Brescia and Rescigno, 2021). However, the mechanism(s) that definitively link perturbations within the gastrointestinal environment to liver health, and ultimately altered brain function, in patients with chronic liver disease have not been established. In contrast, a plethora of studies have evaluated the role of the microbiome in modulating primary CNS diseases (Cryan et al., 2020). Reviewing some these studies may provide a window of understanding as to how perturbations in the gut can affect liver and brain health outcomes.

The host microbiota has been implicated in various CNS diseases including Multiple Sclerosis, Parkinson’s disease, autism, anxiety, and depression (Cryan et al., 2020; Nikolova et al., 2021; iMSMS Consortium, 2022). Microbiome dependent host innate, and adaptive immune responses have been extensively studied in the setting of pre-clinical models of neuroinflammation. These studies have linked changes in the microbiome to changes in host intestinal signaling that are in turn associated with the development of pathogenic/inflammatory T cells that can exacerbate CNS inflammation in preclinical models of MS and autism (Sano et al., 2015; Kim et al., 2017; Lee et al., 2020; Schnell et al., 2021). Similarly, gut microbiota-driven changes in local intestinal B cell cytokine production has also been implicated in attenuating CNS viral infections (Ramakrishna et al., 2019). In addition, the host microbiome can affect local CNS immune responses by altering systemic cytokine or metabolite production (further discussed below). For example, microglial cells are tissue resident immune cells within the CNS that are important in CNS inflammatory conditions (Prinz et al., 2019). The development of microglia and their ability to mount an inflammatory response is dependent on the presence of the host of microbiome (reviewed elsewhere) (Mossad and Erny, 2020). The central role of astrocytes in inhibiting CNS inflammation was also suggested to be mediated by a specific subset of LAMP1^+^TRAIL^+^ astrocytes that can induce T cell apoptosis within an inflammatory environment (Sanmarco et al., 2021). Interestingly, the function of this subset of astrocytes is dependent on meningeal NK cells producing interferon gamma in a microbiome dependent manner (Sanmarco et al., 2021). These studies link aberrant systemic immune responses to the host gut microbiome and highlight its downstream effect on CNS disease. It is therefore possible that a similar complex avenue of communication exists in patients with chronic liver disease who can have debilitating psychological symptoms without overt CNS inflammation.

Efforts to manipulate the microbiota to change liver disease-associated brain dysfunction and altered behavior has been trialed by our group in a pre-clinical model in which we studied the effects of the probiotic mixture VSL #3 on sickness behavior development in mice with cholestatic liver injury. Oral gavage with VSL#3 resulted in decreased systemic pro inflammatory cytokine levels associated with attenuation of sickness behavior development (D’Mello et al., 2015). Moreover, manipulation of the microbiome has also been attempted in patients with liver disease and alcohol use disorder, and those with hepatic encephalopathy, via fecal microbial transplant (Bajaj et al., 2017, 2021). Results from these pioneering clinical trials showed benefit in terms of a decrease in alcohol craving and improvement in cognition (Bajaj et al., 2017, 2021).

## Neural pathways

The liver is innervated by both splanchnic and afferent vagal nerves that closely associate with portal veins, hepatic arteries, and the biliary system (Mizuno and Ueno, 2017). The hepatic autonomic nervous system plays a central role in sensing extracellular osmolality, glucose, lipids, and maintains metabolic homeostasis (Jensen et al., 2013). Vagal innervation of the liver may provide a direct means for changes within the liver environment to be communicated to the CNS. Specifically, studies evaluating intraperitoneal injections of lipopolysaccharide in rodents found expression of early markers of neuronal activation in the vagal afferent nerve primary cerebral projection area, the nucleus tractus solitarius, and in secondary cerebral projection areas (Wan et al., 1993). This observation suggests that peripheral signaling from the liver to the CNS can be mediated via the vagus nerve. However, it is important to highlight that neural signaling pathways alone are unlikely to be solely responsible for some of the CNS manifestations observed in patients with chronic liver disease. In particular, patients with chronic cholestasis (primary sclerosing cholangitis or primary biliary cholangitis) often experience persistent chronic fatigue after liver transplantation, where the transplanted liver is completely denervated (van Ginneken et al., 2010; Wunsch et al., 2021). This suggests that additional non-neural pathways also likely mediate CNS generated symptoms. Interestingly, recent work has highlighted a complex liver-brain-gut neural arc whereby liver afferent vagal nerves, after indirectly sensing luminal contents, signal to the nucleus tractus solitarius of the brainstem, leading to maintenance of gut T-regulatory cells via vagal parasympathetic nerves and enteric neuron signaling (Teratani et al., 2020). This neuroimmune circuit further adds to the complexity of gut-liver-brain homeostasis by raising the possibility of microbiome dependent changes in host neural signaling which affects the CNS and subsequently host immune function. Consistent with these findings, a recent report highlighted the gut microbiota as affecting gastrointestinal motility via extrinsic sympathetic activation and signaling through the brainstem (Muller et al., 2020). These studies showcase the potential of complex neural pathways that, at the very least, represent an adjunct means of relaying host peripheral microbial and immune signals to the CNS in the setting of liver disease to drive adverse symptom generation commonly encountered in chronic liver disease patients.

## Metabolites

The gut microbiome can produce a variety of molecules capable of communicating with host cells. Preclinical studies have evaluated the role of some of these molecules with regards to how they may potentially act to communicate with the CNS. One such family of molecules are short chain fatty acids (SCFA). SCFA are produced by gut microbes acting on undigested food fiber and can interact with a variety of host receptors, including monocarboxylate transporter 1, sodium-dependent monocarboxylate transporter 1, free fatty acid receptors (FFARs), an orphan G protein coupled receptors (Dalile et al., 2019). There have been studies in both patients and animal models that have evaluated whether SCFA produced in the gut can reach the CNS. Studies utilizing carbon tracing showed that, although blood-borne SCFA could in theory cross the blood brain barrier, the total concentration of SCFA within the CNS and cerebral spinal fluid is minuscule (Song et al., 2009; Kim et al., 2013).Therefore, whether these low levels of SCFA can impact local CNS function has yet to be evaluated clinically, and the role of SCFA in CNS dysfunction associated with chronic liver disease remains unclear. There is however pre-clinical evidence of SCFA affecting the CNS. One pre-clinical study found that the SCFA butyrate can attenuate the development of endotoxin-induced sickness behaviors in mice; a finding attributed to a concurrent reduction in proinflammatory cytokine levels (i.e., IL-1β and TNF α) in the brain (Sherry et al., 2010).

Alternatively, SCFA may enter the CNS through the blood brain barrier and be utilized as a reagent in the local production of neurotransmitters. Specifically, a preclinical study showed that administration of isotope labeled SCFA acetate accumulated within the hypothalamus, impacting the GABA neuroglial cycle and leading to increased central production of GABA which enhanced central appetite regulation (Frost et al., 2014). This same mechanism could occur with other neurotransmitters, whereby precursor molecules produced by host gut microbiota could enter the CNS via the systemic circulation and change local production of neurotransmitters to impact CNS function. Further studies are needed to better evaluate this in patients with chronic liver disease and disease-associated behavioral symptoms.

SCFA could also potentially signal the brain through an intermediary compartment, including peripheral neurons and/or by activating the host immune response, to impact the CNS. The SCFA Receptor FFAR3 has been described as being expressed by sympathetic vagal neurons, as well as by neurons of the enteric nervous system (De Vadder et al., 2014; Nøhr et al., 2015). Vagal signals are relayed in the brainstem, where gut vagal afferents synapse onto neurons in the (nucleus tractus solitarius) NTS. From the NTS the neural signal can be relayed to the parabrachial nucleus, locus coeruleus, hypothalamus, amygdala, bed nucleus of the stria terminalis and ventral tegmental area (VTA) to alter reward, motivation, emotion, and stress responses; changes that can ultimately affect behavior (Fülling et al., 2019). We can therefore hypothesize that this may be another avenue through which an altered microbiome in the setting of chronic liver disease can impact CNS function in patients afflicted with symptoms such as depression, fatigue, and social withdrawal.

Interestingly, the host gut microbiota (either *in vitro* or *in vivo*) can produce neurotransmitters and neuropeptides, including dopamine, norepinephrine, gamma-aminobutyric acid (GABA), acetylcholine, and predominately via communication with gastrointestinal enterochromaffin cells, serotonin (Strandwitz, 2018). It is still unclear however, whether the local gastrointestinal production of these neurotransmitters have any extra-intestinal impact, including on the liver, and whether these neurotransmitters can penetrate the blood brain barrier to impact CNS neuronal signaling (Jameson et al., 2020). Further studies are needed to better understand the cellular context and overall organ impact of these microbial derived neuropeptides.

The complex potential role of gut microbiota mediating CNS disease outcomes was discussed above. From a metabolomic standpoint, host microbiota-derived SCFA have also been implicated in the control of microglia function and maturation via the FFAR2 receptor (Erny et al., 2015). We and others have previously shown that microglia are important in mediating sickness behavior development (including in the setting of liver disease), inflammatory CNS conditions and neurodegenerative diseases (D’Mello et al., 2013; Abdel-Haq et al., 2019). From an adaptive immune standpoint, gut luminal microbes, and metabolites, including SCFA impact T lymphocyte immune responses. Specifically, both microbes and SCFA promote differential T lymphocyte function in a preclinical model of multiple sclerosis (Haghikia et al., 2015; Lee et al., 2020), and affect interleukin 17 producing gamma-delta T lymphocytes in a preclinical stroke model (Benakis et al., 2016). These studies evaluated primarily inflammatory CNS conditions. Therefore, whether metabolite-responsive adaptive immune responses have an important role in driving fatigue and other behavioral symptoms observed in the setting of chronic liver disease is yet to be determined.

**Figure 1:**
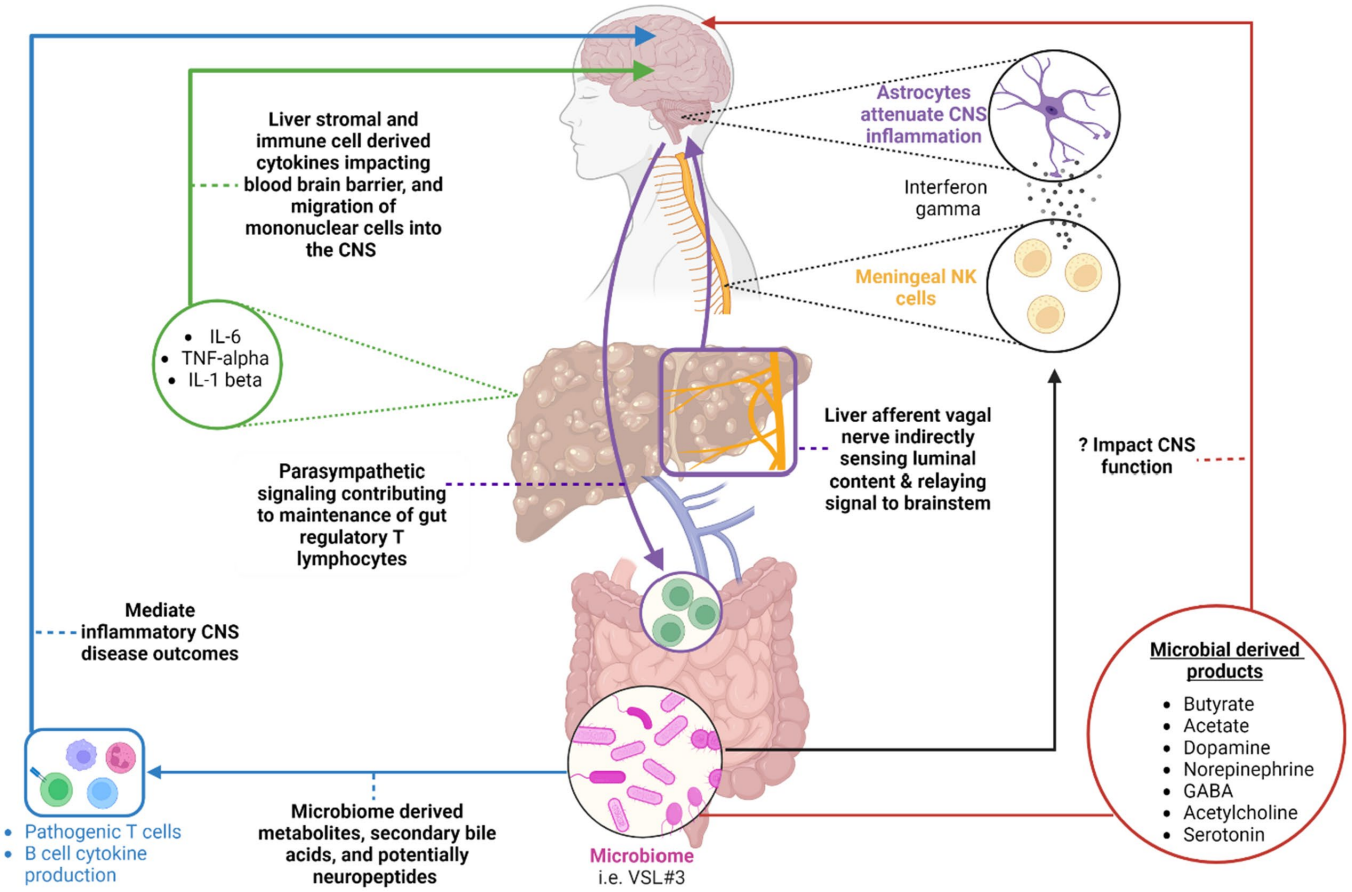
A summary of the various avenues within the gut-liver-brain axis that may be perturbed in patients with chronic liver disease. Original references for these avenues are found in the main body of the text. (Blue) highlights the impact of microbial derived metabolites, secondary bile acids, and potentially neuropeptides on gut environment and systemic adaptive immune cell function in the setting of inflammatory CNS conditions. (Red) Highlights various microbial-derived products including short chain fatty acids [butyrate, acetate, etc.] and neurotransmitters that may potentially impact CNS function in the setting of chronic liver disease. (Purple) A circuit whereby hepatic afferent vagal nerves indirectly sense luminal contents and relay resulting neural signals to the brain stem. The brain stem in turn, via parasympathetic efferent neurons, impacts the maintenance of T regulatory cells in the intestine. (Black) An additional pathway whereby meningeal NK cells produce interferon gamma to impact astrocyte function with a resulting modulation of CNS inflammation. The ability of the NK cells to produce interferon gamma is dependent on the microbiome. (Green) Liver immune and stromal cell derived cytokines can impact the blood-brain barrier function and recruitment of mononuclear cells to the CNS. This can enhance sickness behavior development in the setting of liver injury. Created with BioRender.com.

Although in this review we describe and classify discrete pathways by which gut microbiome-activated systemic perturbations may lead to changes in the CNS, it is likely that these avenues intersect and connect as a complex network to signal to the CNS, and vice versa. For example, a recent study evaluated the complex interplay between the host gut microbiome, metabolic function, and neural function with regards to its effect on the motivation for exercise. Specifically, the microbiota-dependent production of endocannabinoid metabolites interacted with TRPV1-expressing sensory neurons in the periphery to subsequently signal the brain to elevate dopamine levels in the ventral striatum, enhancing the motivation for exercise (Dohnalová et al., 2022). It is therefore possible that changes occurring in the gut microbiome, its constellation of produced metabolites, and neuronal signaling that occurs during chronic liver disease can in turn signal to the CNS to promote the development of detrimental symptoms such as fatigue, social withdrawal, cognitive impairments, and mood disorders (Figure 1).

## Liver-brain axis in clinical disease

The neurological complication often associated with end stage liver disease is hepatic encephalopathy (HE). The subtypes of HE, its complex pathophysiology, the known roles of neurotoxic metabolites, serum ammonia, and bile acids have been reviewed and discussed elsewhere (Rose et al., 2020). In this section, we will focus on outlining the clinical neurological abnormalities that have been reported in the setting of different chronic liver disease processes.

### Viral hepatitis

The effects of chronic viral liver infection on global neurocognition are not well understood. In a cross-sectional study, close to 200 patients with chronic Hepatitis B infection (HBV), Hepatitis C infection (HCV), and treated HCV with sustained virological response were evaluated. Neurological exams, neuropsychological testing, and bloodwork found HBV was associated with impaired language and executive function. HCV was associated with executive function, psychomotor speed, memory, and attention deficits. Interestingly patients that achieved SVR (i.e., viral cure) with HCV treatment did not show improvements in their performance on neuropsychological testing, contrary to what had been reported prior (Barbosa et al., 2017; Shehata et al., 2022; Tan et al., 2022). The authors of this study postulated that differences in attention deficits may relate to systemic inflammation, but no mechanistic work was done to support this suggestion (Tan et al., 2022). Findings from this study are consistent with other studies that noted differences in anxiety, depression, attention and memory in non-cirrhotic HCV patients associated with brain grey matter changes noted on Magnetic Resonance Imaging (MRI) and MRI spectroscopy (Weissenborn et al., 2004). Although low levels of HCV can be detected and may replicate within the CNS (Radkowski et al., 2002; Fishman et al., 2008), the impact of this on global CNS function is not clear. Of interest, a study from Taiwan found an epidemiological association between HCV infection and Parkinson’s Disease and illustrated HCV-mediated dopaminergic neuron death using an *in vitro* rat neuron co-culture system (Wu et al., 2015). Similarly, others have reported an association between dementia and HCV but not HBV infection (Choi et al., 2021). Again, these studies highlight interesting clinical associations but the mechanisms governing these outcomes remain elusive.

### Autoimmune and cholestatic liver disease

Neurological symptoms including fatigue, memory impairment, and sleep disturbances have been reported in patients diagnosed with autoimmune hepatitis, Primary Biliary Cholangitis (PBC), and Primary Sclerosing Cholangitis (PSC) (Dyson et al., 2015; Zenouzi et al., 2018; Jones et al., 2023). The underlying mechanism governing development of these symptoms is not fully understood, and treatment options are currently an area of unmet need. In addition to the role of the host immune response highlighted prior, other clinical avenues have been investigated by others. In the setting of PBC, daytime somnolence has been suggested by one group to be related to altered intracortical inhibition and intracortical facilitation detected via trans cranial magnetic stimulation (McDonald et al., 2010). Our group has utilized MRI to evaluate both structural and functional CNS changes occurring in the setting of PBC. We found that PBC patients exhibit reduced thalamic volume along with reduced anterior insula activity; regions of the brain that are important in mediating one’s sense of the body’s internal state (Mosher et al., 2019). Others using similar imaging modalities have reported no structural changes in the brain of PBC patients suffering with fatigue (Zenouzi et al., 2018). Alternatively, altered cerebral vascular resistance and abnormal cerebral autoregulation stemming from CNS and peripheral autonomic dysfunction has been postulated by some to drive the development of these cognitive symptoms in PBC patients (Newton et al., 2008; Hollingsworth et al., 2010). Another study utilizing brain MRI testing and MR spectroscopy have found similar perturbations in brain photon density and increase in metabolites including N-acetyl-aspartate, and choline in regional white matter regions in patients with either AIH, HCV or PBC, raising the possibility of shared pathophysiological mechanisms across these conditions. However in this study, there were no correlations between MRI/MR spectroscopy findings with neuropsychological dysfunction (Reichardt et al., 2022).

### Wilson’s disease

Wilson’s disease (WD) is an inherited condition resulting from impaired excretion of copper into the biliary tract by hepatocytes harboring mutations in transmembrane transporter protein, ATPase copper transporting beta. Excess copper in hepatocytes is released into the bloodstream where it can then lead to the dysfunction of other systemic organs including the CNS (Scheiber et al., 2017). Neurological manifestations of WD can be quite variable across different patients with symptoms including dysarthria, movement disorders, rigid dystonia, seizures, Parkinsonism, psychiatric manifestation and sleep disorders (Roberts et al., 2022). Imaging studies evaluating CNS involvement in WD have highlighted atrophy or volumetric decrease in subcortical nuclei regions, diffuse white matter, and some grey matter zones (Song et al., 2021). These changes concerningly were suggested by one group to be found in WD patients lacking neurological symptoms (Rędzia-Ogrodnik et al., 2022), highlighting how structural changes precede clinical manifestation that in part may explain the heterogeneity in CNS symptomology in these patients. In addition to the structural changes, excess iron and manganese deposition also occurs in the brains of WD patients, though their role in CNS disease development and progression is unclear (Klos et al., 2006; Litwin et al., 2013).

### Non-alcoholic fatty liver disease

Non-alcoholic fatty liver disease (NAFLD) is currently and will continue to be a major driver of chronic liver disease in the years to come. The neurological outcomes associated with this liver condition span a wide spectrum in part due to shared risk factors for vascular CNS diseases including hypertension, atherosclerosis, dyslipidemia, insulin resistance and diabetes (EASL-The Home of Hepatology, 2016). Given this, neurological complications of NAFLD can overlap with those experienced by cardiovascular patients and include transient ischemia attacks, ischemic stroke (Alexander et al., 2018), vascular dementia (Shang et al., 2022), and altered cerebral blood flow (VanWagner et al., 2017). However, studies have also found that NAFLD on its own is a risk factor for cognitive impairment (Seo et al., 2016), as highlighted in a study that found an association between NAFLD and smaller total cerebral brain volume that was independent of visceral adipose tissue and cardiovascular risk factors (Weinstein et al., 2018). Others have also interestingly reported an association of NAFLD with altered executive and frontal lobe function, depressive mood, anxiety, and apathy (Moretti et al., 2019). Further linking liver function and brain health, in a large population-based study of over 3,400 patients without dementia or prior stroke, NAFLD was found to be associated with alterations in white matter microstructural integrity, lower cerebral blood flow, and lower brain perfusion as evaluated using MRI (Yilmaz et al., 2023). Animal studies have found similar changes to cerebral brain volume and suggest altered metabolism may be contributing to this. Specifically, in a murine diet model of NAFLD, proton magnetic resonance spectroscopy found changes in cerebral volume in parallel with alterations in thalamic levels of total N-acetyl aspartate, total creatine, total choline, and taurine; metabolites that are involved in energy metabolism and brain function. These structural changes mirror what has been noted in NAFLD patients and the authors of this study postulated that the metabolomic changes observed are secondary to increased levels of proinflammatory cytokines, perturbations in endothelial cell function, and/or changes in brain microglial cell function that ultimately impact brain function; something that we have also observed in our prior studies using cholestatic mice (Kerfoot et al., 2006; D’Mello et al., 2015; Nucera et al., 2022).

## Concluding remarks

Behavioral symptoms, including fatigue, social withdrawal, and mood disturbances, are commonly encountered in the clinical setting in patients with chronic liver injury. These symptoms have a detrimental effect on patient quality of life (Mells et al., 2013; Cheung et al., 2016). Currently, therapeutic options to ameliorate these symptoms are limited, driven in large part by an incomplete understanding of the complex pathways that link the gut and chronic liver injury/inflammation in driving altered CNS function. In this review, we have summarized current understanding and discussed potential candidate pathways that mediate this body–brain communication. These pathways likely include an altered immune response (either systemically or within the CNS), altered gut metabolite production, differential peripheral and central neuron function, and complexities of an impact of a perturbed host gut microbiome on host CNS function. Further pre-clinical and clinical work is needed to better characterize both local CNS and systemic changes occurring in subjects with chronic liver disease as they relate to behavioral symptoms. An improved understanding of these complex pathways and their intertwined network of interactions will provide us with new therapeutic avenues for the treatment of these debilitating symptoms.

## Author contributions

All authors listed have made a substantial, direct, and intellectual contribution to the work and approved it for publication.

## Conflict of interest

The authors declare that the research was conducted in the absence of any commercial or financial relationships that could be construed as a potential conflict of interest.

## Publisher’s note

All claims expressed in this article are solely those of the authors and do not necessarily represent those of their affiliated organizations, or those of the publisher, the editors and the reviewers. Any product that may be evaluated in this article, or claim that may be made by its manufacturer, is not guaranteed or endorsed by the publisher.
